# Designing Videos With and for Adults With ADHD for an Online Intervention: Participatory Design Study and Thematic Analysis of Evaluation

**DOI:** 10.2196/30292

**Published:** 2021-09-14

**Authors:** Eivind Flobak, Emilie Sektnan Nordby, Frode Guribye, Robin Kenter, Tine Nordgreen, Astri J Lundervold

**Affiliations:** 1 Department of Information Science and Media Studies University of Bergen Bergen Norway; 2 Division of Psychiatry Haukeland University Hospital Bergen Norway; 3 Department of Clinical Psychology University of Bergen Bergen Norway; 4 Department of Biological and Medical Psychology University of Bergen Bergen Norway

**Keywords:** participatory design, ADHD, online intervention, video, therapeutic content, stigma, attention deficit hyperactivity disorder, design, participatory, intervention, experience, mental health

## Abstract

**Background:**

Adults with attention deficit hyperactivity disorder (ADHD) represent a heterogeneous group with both strengths and difficulties associated with the diagnosis. An online intervention attuned to their needs may improve their everyday functioning. When designing online interventions, it is important to adapt the therapeutic content to the values and needs of the target group.

**Objective:**

This paper describes and evaluates a participatory process used to produce content for an online intervention for adults with ADHD by producing video vignettes clarifying core training principles grounded in the participants' everyday experiences.

**Methods:**

We report on the qualitative data from 2 research phases: the design and evaluation of video vignettes for an online intervention. In the first phase, 12 adults with ADHD, 2 clinicians, and 2 research assistants participated in the production of video vignettes for the online intervention. In the second phase, participants (n=109) gave feedback on the videos as part of a clinical trial of the intervention. A subgroup (n=7) was interviewed in-depth regarding their experiences with the videos. The qualitative data were analyzed using thematic analysis.

**Results:**

In the first phase, the participants with ADHD contributed with experiences from challenging everyday situations. In the process, we navigated between therapeutic principles and the participants' experiential perspectives to create content relevant and consistent with the target group's values and experiences. In the second phase, we identified 3 themes related to the participants' experiences and interpretation of the video vignettes: (1) recognition of ADHD-related challenges, (2) connection with the characters and the situations, and (3) video protagonists as companions and role models for change.

**Conclusions:**

A participatory design process for designing online mental health interventions can be used to probe and balance between the therapeutic principles defined by clinicians and the participants’ experiences with mental health issues in the production of therapeutic content. In our study, the inclusion of video vignettes in an online intervention enabled a contextualized and relevant presentation of everyday experiences and psychosocial factors in the life of an adult with ADHD.

**Trial Registration:**

ClinicalTrials.gov NCT04511169; https://clinicaltrials.gov/ct2/show/NCT04511169

## Introduction

### Background

Interactive technology has the potential to enhance mental health treatments by improving their access, affordability, and effectiveness [1]. When designing online psychological interventions, emphasis on engagement and relevance for the users is a key element [2-5]. To achieve this engagement, a match between the intervention’s content and the person’s psychosocial and behavioral characteristics is essential [6], as it can lead to higher satisfaction and, thereby, higher engagement and better outcomes [7]. Moreover, considering the perspective of people with mental illnesses has been recognized as a way to ensure that the content is relevant and consistent with their values [8-10]. Previous studies have, therefore, employed person-centered approaches when designing content for online interventions, such as user-centered [4,9] and participatory [11] design methods.

In general, online interventions with guidance from a supporter (eg, a therapist) result in higher engagement and improved treatment outcomes when compared to unguided interventions [7,12]. This means that the social interaction with a supporter is a primary contributing factor to the success of the intervention [13]. Unguided interventions are still commonly chosen when searching for low-cost therapeutic resources for people with limited access to mental health care services [14]. Unguided interventions do, however, place new demands on how therapy should be presented to clients [7]. Without a relationship between client and a therapist, an unguided intervention regimen requires content that is clearly recognizable to the target group [7]. Generally, people expect a user experience (see [15]) that caters to their needs and desires related to the technology and the context of use [16]. In a similar vein, Borghouts et al [7] found that people are more likely to engage with an online intervention if they experience “the program to be useful and a good fit for them” [7].

Adults with attention deficit hyperactivity disorder (ADHD) are expected to benefit from an unguided online intervention, as it is a low-cost, easily accessible, and flexible alternative. This is related to the disorder’s frequency, with an estimated prevalence of 2%-7% [17]; heterogeneity; and characteristics. ADHD in adults is defined by core symptoms of inattention, impulsivity, and hyperactivity [18] and persistence of symptoms from childhood [19,20]. They frequently report problems related to self-regulation [21,22] and commonly face several everyday challenges [23,24]. Pharmacological treatment is the first choice of intervention for this disorder, but many adults commonly ask for nonpharmacological alternatives [25]. Still, most individuals with ADHD do not have access to psychological treatment in adulthood [25]. Technology may, thus, represent an opportunity to support adults with ADHD [26].

Qualitative studies of everyday experiences of people with ADHD provide insight into the lived experiences of having ADHD and demonstrate how the term “people with ADHD” refers to a heterogeneous group and that there are several positive traits attributed to the disorder. In a study by Holthe and Langvik [27], a group of women with ADHD reported positive attributes such as “high energy, creativity, determination, ability to get easily interested and excited about new things, adventurousness, and willingness to take risks” [27]. In another study, diagnosed adults reported curiosity and hyperfocus as positive attributes of their disorder [28]. In a study by Wiklund et al [29], entrepreneurs with ADHD defined their impulsivity and hyperfocus as the major drivers of their entrepreneurial action. However, the participants in the study by Holthe and Langvik [27] also described challenges in everyday life, including poor time management of daily plans and procrastination of tasks that lead to a sense of constantly being behind schedule. Adults with ADHD who were diagnosed in adulthood (ie, growing up with undiagnosed and untreated ADHD) reported that they were ridiculed and received negative comments from their peers and families during childhood, such as being referred to as “stupid, lazy, and disruptive” [30]. The participants in this study also reported that they coped with the negative feedback by either accepting the remarks as accurate representations of their character or by ignoring the remarks altogether to evade feelings of low self-esteem [30].

Public stigma causes additional challenges for people with ADHD [31-38]. Stigma refers to socially situated characteristics of a person that enable the dehumanizing processes of prejudice, stereotypification, and discrimination [39]. Stigma toward people with mental disorders is common, imposing social misfortunes on them [35,40]. In adults with ADHD, public stigma is commonly converted into self-stigmatization, a tendency to internalize negative public attitudes and beliefs of one’s characteristics [41,42]. Consequently, they may experience decreased quality of life, discontinuation of treatment, social isolation, and low self-esteem and self-efficacy [37,40]. Young et al [30] showed that adults with ADHD who learned to emotionally accept their diagnosis and themselves, ultimately, had enhanced self-esteem and received improved support from people close to them. Similarly, in a study with adolescents with ADHD reporting retrospectively on self-stigmatization [43], some participants had gained resilience against negative judgments through the confidence in themselves and the acceptance by those close to them.

Disclosures of mental health in social media have challenged public stigma and empowered people with mental health issues [44-48]. Visual stories have particularly influenced the connection and empowerment of those with lived experiences similar to those disclosed [46,49,50]. Feuston and Piper [51] focused on how people share narratives of their mental illness on social media, through the analytical framework of small stories [52], emphasizing their importance in understanding mental illness from an experiential point of view. This type of health communication differs from the prescriptive, fact-based style of clinicians’ expertise since patients’ expertise is characterized by a narrative style of coping with day-to-day challenges by trial and error [53]. Moreover, it is recognized that seeing how others handle everyday situations can contribute to learning of new behavior (eg, Bandura’s social cognitive theory [54,55]). Studies on how people disclose their struggles with mental illness on social media and the associated engagement with these media should have implications on the content design of online interventions. Narrative communication has also been used as a tool in health behavior change [56], recently by the use of short videos in an intervention for reducing stigma towards people with schizophrenia [57,58] and increasing treatment seeking for depression [59]. In these videos, schizophrenia is presented “with a human face rather than as a ‘brain disease’” [57] and show people with severe mental illness being capable of working and having meaningful relationships. Addressing the experiences of adults with ADHD through an everyday lens [51] was, therefore, our objective when designing therapeutic content that is relatable and relevant to the target group.

### Aims and Objectives

This study goes beyond the focus on the management of core symptoms and considers everyday experiences of adults with ADHD in the design of an online intervention. We explored how a participatory approach can ensure that the participants’ perspectives are included in the design of psychoeducative therapeutic content in mental health interventions. With this aim, we directly included adults with ADHD in the design process to empower their perspective through active participation. In this study, their experiences were ultimately represented as video vignettes in an online intervention, which aimed at communicating the therapeutic principles and coping techniques. A further aim of this study was to explore how participants in an online intervention experienced and made sense of the video vignettes.

## Methods

### Study Design

The qualitative research presented in this paper was conducted in 2 phases: (1) a participatory process for the design of video vignettes as psychoeducative, therapeutic content for an online intervention for adults with ADHD; (2) the collection and analysis of qualitative feedback on the video vignettes during and after a clinical trial of the online intervention.

### Study Context: “MyADHD” Online Intervention

The intervention addressed in this paper, MyADHD, was designed as a modular web-based course. Once a module was opened, the participant could browse freely within all the available modules and revisit content as they saw fit. MyADHD is comprised of 7 modules representing different topics (Introduction, Breathing, Stop, Emotions, Planning, Acceptance, and Conclusion). Each of the first 6 modules included psychoeducative information, skill-building exercises, and coping techniques for everyday challenges for adults with ADHD. Additionally, each module featured 2 video vignettes that explained the use of coping techniques and contextualized their suitability and application.

The process of designing the intervention was established on the *person-based approach* [4,60]. In designing the program’s content, the method was extended by including adults with ADHD as co-designers through a 3-year participatory process. The intervention builds on the therapeutic principles of cognitive behavioral therapy (CBT), Goal Management Training, and dialectical behavioral therapy (DBT). The intervention aims to improve everyday functioning in adults with ADHD by providing psychoeducation, skill-building exercises, and coping techniques.

### Phase 1: Participatory Design Study

#### Participatory Design

We position our design process as participatory design [61-64]. According to Ehn [61], this approach to designing technology is strategically motivated from 2 different levels: (1) a democratic commitment to ensure the end users’ representation in the processes of making technology that concerns them [61,64] and (2) “the importance of making the participants’ (*sic*) tacit knowledge come into play in the design process” [61]. Our study was essentially concerned with the second level: the engagement of those with an ADHD diagnosis in the design process.

#### Participants

A total of 12 participants were recruited from a local division of a nationwide, voluntary patient organization for people with ADHD and their relatives. The participants were defined as experts by experience. Originally, 3 participants were recruited by convenience sampling. When the participatory design process reached a stage where we were designing content, the number of participants was increased to 12 to improve the representation of the diversity in the target group. This further recruitment was done by combining convenience and snowball sampling. Participants were given a gift certificate of 200 NOK (US $24) for their contribution to the design process.

The team also included 2 clinical psychologists (clinicians) and 2 clinical psychology students employed as research assistants in this study (assistants). These participants had a facilitating role in the design process.

#### Workshops

To understand preferences and needs for web-based services, the research team invited a small group of adults diagnosed with ADHD (n=3) to participate in meetings and workshops. In an early meeting, they were asked to discuss and evaluate the benefits of already available information (eg, social media, webpages, YouTube), and to present their evaluations at the next meeting with the research team. Videos on social media were their preferred format, exemplified by videos presented by a YouTube ADHD health vlogger (eg, “How to ADHD” on YouTube [65]). The participants emphasized the light-hearted, self-deprecating humor displayed on the YouTube videos and described the importance of not being talked down to by someone in a “superior position” (the participants’ quote). From this and other follow-up discussions, the research team decided to design short video vignettes as the core content of the online intervention.

The participatory design process was predominantly performed through workshops where the different parties (ie, participants, clinicians, and assistants) discussed and committed to shared goals by the methods of ideation and co-design. The workshops were organized by the clinicians and the assistants and were documented by note-taking and archiving workshop materials. In the workshops, the facilitators asked open-ended questions to engage participants in discussions of how ADHD impacted their everyday lives and how they coped with challenging situations.

### Phase 2: Evaluation

#### Overview

The evaluation presented in this paper was done as part of a clinical trial conducted between May 2020 and October 2020. Participants were expected to complete the MyADHD intervention over 7 weeks, with 1 new module becoming accessible every week regardless of the participants’ progression. The intervention was unguided, with the only supporter interaction being a pretrial telephone screening and an automated SMS text messaging notification each time a new module was released. The criteria for inclusion in the study were (1) age 18 years or above at inclusion and (2) a self-reported diagnosis of ADHD. Exclusion criteria were (1) current self-reported diagnosis of a severe psychiatric disorder and (2) other ongoing psychological treatment. The patient organization distributed a recruitment website on their Facebook Pages in May 2020, 2 months into the global COVID-19 outbreak. The study was approved by The Regional Committee for Medical Research Ethics of Western Norway (2020/90483).

#### Participants

The clinical trial included 109 participants, 88 of whom (80.7%) identified as women and 21 (19%) as men. Participants’ ages ranged from 22 to 62 years (mean 36 years, SD 9 years). More than half of the participants (62/109, 56.9%) reported having completed university- or college-level education. At the time of inclusion, 67.9% (74/109) reported being employed or students, and 32.1% (35/109) were on sick leave or unemployed or received a disability pension. The participants’ pretrial scores on the Adult ADHD Self-Report Scale (ASRS) [66] are shown in Table 1. More specifically, 1 participant scored below threshold (17 points) on both the inattention and hyperactivity-impulsivity subscale, while 108 (99.1%) participants scored above the screening threshold on one of the subscales [67]. Informed consent was signed by the participants upon logging into the online intervention website for the first time. All participants were given a gift certificate of 400 NOK (US $48) upon completion of the study’s posttrial questionnaires.

**Table 1:** Pretrial Adult ADHD Self-Report Scale (ASRS) scores for 109 participants.

Scale	Results, mean (SD)
Total ASRS scale	49.6 (9.2)
ASRS Inattention subscale	26.8 (4.6)
ASRS Hyperactivity-impulsivity subscale	22.8 (6.3)

#### Online Intervention Procedure and Data Collection

For each module, apart from the first Introduction and seventh Conclusion modules, participants watched 2 video vignettes: (1) a version of a situation where the protagonist experiences everyday challenges associated with the topic of the MyADHD module and (2) a version of the same situation where the protagonist successfully handles the situation with the use of a technique featured in the MyADHD intervention. Example videos are available in Multimedia Appendices 1-4. Descriptions of all videos are available in Table S1 in Multimedia Appendix 5.

At the end of each module, participants were asked to rate their satisfaction with the module’s content and to provide qualitative feedback about their opinion on the module. The following 3 questions were asked, with an open-ended text input field for each question: (1) “What did you like best or what did you find most useful in this module?” (2) “What did you miss or what disappointed you in this module?” and (3) “In the module, you saw some videos. How would you evaluate them?” The seventh and final module was a summary of all the material and asked the participant for the key takeaways of each module. Lastly, participants completed a posttrial questionnaire on the entire intervention. Across 6 modules, 79 participants (79/109, 72.5%) provided feedback on the videos, with a total of 275 responses and 2850 words. See Table 2 for further descriptive statistics about data referring exclusively to videos (question number 3).

**Table 2:** Breakdown of qualitative feedback gathered from the module evaluation within the online intervention.

Feedback item	Number of words, mean (SD)	Responses, n
Intro video	10.5 (10)	51
Breathe videos	9.5 (13.8)	61
Stop videos	12.8 (17.1)	49
Emotion videos	13.2 (20.1)	35
Planning videos	8.3 (14.4)	43
Acceptance videos	6.5 (7.2)	36

All data recorded for this study, including the interview transcripts, were securely stored on a hospital research server. All participants were free to withdraw their participation and have their data removed at any time.

#### Data Collection: Interviews

In a posttrial survey, participants were asked to be interviewed in-depth regarding their experience with completing the intervention. The inclusion criterion was to live in the vicinity of the hospital that ran the main study. To this petition, 20 participants responded positively, and 13 did not qualify for inclusion due to their geographic location. Among the 7 interviewees, 6 self-identified as women and 1 as a man, and they were aged between 27 years and 47 years (mean 35 years, SD 8 years). Participants were interviewed for an average of 1.5 hours at 1-5 weeks after completing their participation in the study.

The interviews followed a semistructured interview guide [68]. The interview focused on the participants’ experience with the videos and included the following open-ended questions: “What do you think of the videos?” “What do you think of the characters?” “What do you think about the situations in the video?” and “How did the videos help you to complete the program?” However, these questions were used more like a checklist for the interviewers rather than a guide, so that the conversation originating from the questions would flow as freely as possible. The interviews were conducted by 1 human-computer interaction researcher (HCI) and 1 research assistant.

#### Data Analysis

The qualitative feedback and the interviews were analyzed following the procedure by Braun and Clarke for reflexive thematic analysis [69,70]. Interviews were transcribed verbatim. Qualitative data from the online intervention were combined with the interview data. Each interview participant was given a gender-neutral pseudonym in the transcripts. Data excerpts from each participant in the online intervention were given a random numerical ID code, with no inclusion of identifying information in the transcripts.

The first author coded the dataset. The following research question was formulated to guide the analysis: “How do participants in the online intervention experience and make sense of video vignettes designed for the intervention?” A semantic, inductive approach was applied to the coding of excerpts, meaning that the coder aimed to understand the participants’ statements as descriptions of their experience. The themes, with their codes and excerpts, were discussed between the first, second, and third authors. Then, codes were rearranged into 3 themes. Upon writing the report of the analysis, quotes that described characteristics and variations of the themes were carefully translated into English.

## Results

### Phase 1: Participatory Design Study

#### Design Process

The clinicians in the research team discussed the therapeutic rationale for designing videos as content for an online intervention, following the practice of defining guiding principles in the person-based approach [4]. They defined 3 core aims: (1) to increase engagement with the intervention using experience-centered content, (2) to clarify and provide examples of core training principles and help participants make connections between the material and their own experiences, (3) to address self-stigmatizing beliefs in adults with ADHD. These aims guided the process of designing videos for the MyADHD intervention.

**Figure 1:**
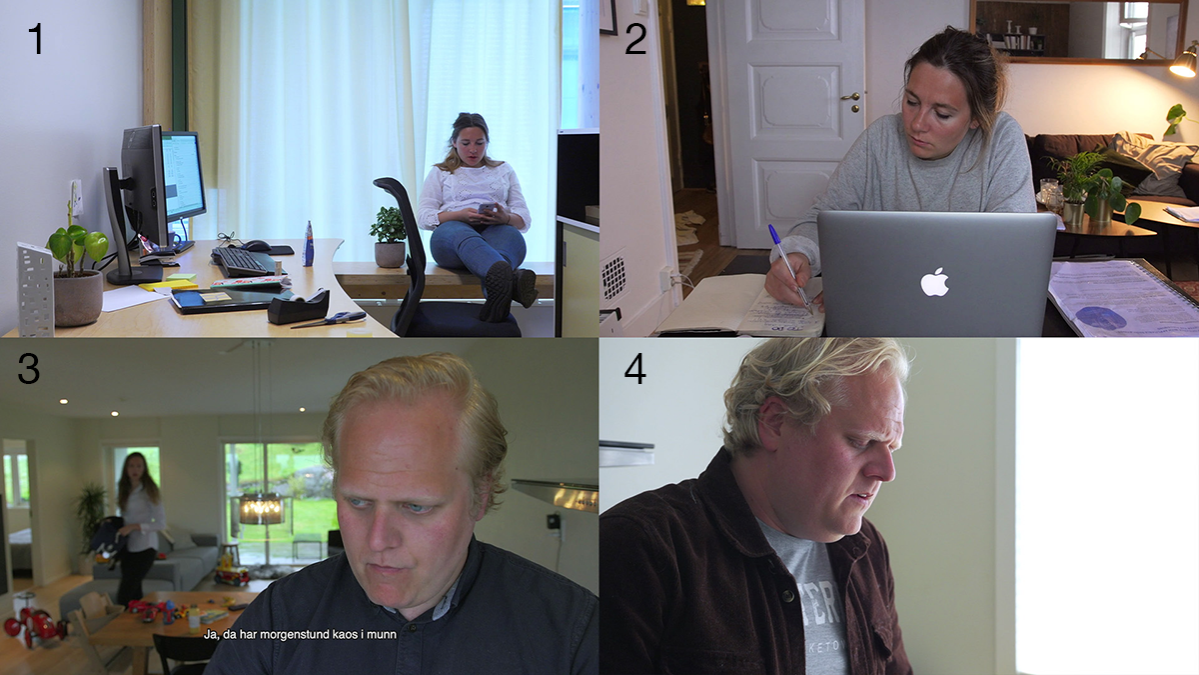
Stills from the video vignettes: (1) Nora procrastinates at her office, (2) Nora is at home doing a planning activity to get her work done during overtime, (3) Erik stresses through the morning routines; his wife and the children's toys are visible in the background, (4) Erik takes a moment to perform a breathing exercise in a moment of distress.

In response to the participants’ request, we made a pilot video intended to display a realistic view of the everyday life experiences of an individual with ADHD. One of the participants expressed an interest in sharing experiences from her everyday life and the impact of ADHD on these activities. Her inclusion in the video production was an effort to resonate with the target group's experiences and values in the video. The participant met with an actress hired for the production. Together, they constructed the character of Nora (Figure 1) as the protagonist of the pilot and wrote the outline based on the participant’s experiences and characteristics. Following this activity, a film production company was hired to adapt the outline into a screenplay and make a video. Next, the participant, a clinician, and an assistant read through the screenplay and approved it after minor revisions to the humoristic tone. During the recording of the video, the participant, a clinician, and an assistant acted as consultants to the actor and the crew to help achieve a result close to the original vision.

The pilot video received positive feedback from health care professionals, students, and a local ADHD patient association. Participants advised the research team to address family life. A second character, Erik (Figure 1), was created. This protagonist represented those adults with ADHD meeting challenges when they combine family, work, and personal life, and a series of videos for the online intervention was produced (Figure 1).

Erik is a family man around the age of 40 years with ADHD. After meeting new demands in his role as a father and husband, he struggles with maintaining his emotional control in stressful situations. Combining work and family life creates situations in which he quickly loses his temper and, thereafter, feels ashamed.

Nora is a woman in her 20s with ADHD. She is a young professional in finance who has a higher education. She struggles with inattentiveness, being easily distracted and forgetful, and often puts off important tasks.

A workshop to generate ideas for MyADHD video vignettes was organized with 12 participants. The participants were asked to share their experiences related to the topics of the modules for MyADHD. The participants were divided into 3 groups, and a facilitator (clinician or assistant) would ask them to describe challenges, daily situations, and coping strategies for each of the topics. In addition, the 2 actors who would play the protagonists attended the workshop, listened to the participants, and asked questions of the participants to better understand their roles.

The participants contributed with experiences from their everyday lives. For example, one participant told a story of being distracted from pressing matters at work by a bird outside her office. Another said that “one time, while at work, I drifted away and searched the web for ‘what is the difference between a magpie and a crow?’” Participants also described difficulties in relation to others, such as not feeling accepted as “a person with ADHD” because their behavior was not within others’ expectations of people with ADHD, or that their challenges were not taken seriously by their acquaintances (eg, “when people tell me that ‘everyone’s like that’ and that ‘everyone forgets things or are unfocused,’ it becomes difficult to accept that I have ADHD, and I need help coping with my symptoms”). Regarding coping strategies, they found breathing exercises to be helpful, but often difficult to follow and complete because their focus could drift away from the activity. Many participants made “to-do lists” daily and set alarms on their phones to remember appointments. For further examples of the participants’ experiences and suggested coping strategies, please refer to Table S2 in Multimedia Appendix 6.

After the workshop, a clinician and the assistants created outlines for video vignettes to accompany the 6 modules of MyADHD. Notes from each of the 3 focus groups were compared and analyzed to extract topics and experiences appropriate for each module. Based on this analysis, outlines for 14 video vignettes were written. These outlines were rewritten into movie scripts in cooperation with the film production team.

During film production, a clinician acted as the consultant and oversaw the process. The film crew applied cinematic techniques of comic timing in acting, pacing in editing, and the use of music to make the videos engaging.

The final videos were shown to the participants who took part in designing the vignettes. Feedback was overall positive, but some found the videos a little too light, because several of the described challenges tend to be experienced simultaneously. Nonetheless, the participants also expressed an understanding for this reduction due to the pedagogical purposes. Overall, the videos were described as recognizable, pedagogical, and easy to follow. They were considered humorous, which was emphasized as a positive trait. The participants reported recognizing themselves in the video and a feeling of being less alone with their challenges. The participants requested additional examples of challenging social situations and how to improve the handling of such situations and more context to understand the challenges and exercises shown in the videos. Subsequently, the clinicians wrote text to introduce the context of each video for the online intervention. The participants approved the final version of the videos.

### Phase 2 Findings: Evaluation

In this section, we present the analysis of qualitative data collected from participants (n=109) who participated in the clinical trial of MyADHD, phase 2 of our study. Common themes included how the participants viewed the characters and their situations and how the videos reflected their own everyday experiences. Furthermore, the participants responded to the videos by showing empathy with the characters, relived their own past experiences in similar situations, and reported that they felt less alone with their challenges and that they used the characters as role models for coping with their everyday problems.

Some participants reported that the videos were the most positive part of the MyADHD intervention. They described them as the main motivation to register for the study, among other things. They declared that watching others experiencing challenges like theirs was self-affirming. Although the use of video was received positively, some participants did not appreciate them: They found the videos boring and too long or too slow, while others found them too short. One participant reported that:

I have not seen them. #impatient

*P* #22

#### Theme 1: “You Can See the Experience” — Recognizing ADHD-Related Challenges

The participants described how the videos depicted situations they recognized from their lives. In addition to the concrete situations, the participants also recognized themselves in the fictional characters of Nora and Erik. In this theme, we unpack how the videos were perceived by the participants as renditions of life with ADHD. We give attention to the participants’ experiences of whether the depictions are perceived as realistic to what it means to have ADHD in everyday situations.

In one of the videos, Nora is distracted at work by various events (eg, a bird outside the window of her office) and must work overtime to complete her economic report. Participant #60 recognized this situation and wrote:

The movies show just the way I feel in everyday life. The hours fly by, and I do not know what I have done.

Certain story elements were recognizable for other participants:

I have several times lost concentration due to birds when I am at work. So that example was a bit funny. :)

*P* #3

Seeing that the presence of a bird outside Nora's office could consume so much of Nora's day was easy to relate to in a humorous manner. The response of Participant #63 further shows that it was possible to recognize oneself in the video while finding it funny, as they simply put it: “[It] was like seeing myself [emoji: Face with Tears of Joy].”

As the videos portrayed relatable characters and situations because of their ADHD symptoms, it is interesting to investigate their level of credibility. In the interview, Charlie explained that seeing the way that the characters handled challenging situations resembled similar experiences they had:

You can see the experience from [the videos], and it is not made in such a way that seems fake. You know...you can watch a feature film and you can see that it is fake. This is realistic.

The content of the video appeared credible to Charlie, and they went on to explain how seeing the videos made it possible to see their own actions in a new light:

[The videos are] done in a way that allows me to see my negative things without it becoming uncomfortable. I do not feel offended.

Elliot shared how the situations displayed in the videos reminded them of concrete experiences and similar situations, such as driving off without noticing or remembering the coffee cup on the roof of the car before it is too late. However, regarding the experience with having ADHD, Elliot was not convinced that the videos gave a credible impression of the felt experience. Elliot said that the videos do not capture the turmoil of having a family and explained how ADHD is heritable and, thus, something their children struggle with as well:

I think [the videos] fall flat. Because it...it does not say anything about the seriousness, in a way, how fierce those [situations] actually are [...] So, I have been sitting in the mornings [after the family is out for work or school] crying. And it is not something I had been doing much before. And I am not afraid of emotions per se, but I am not used to crying. But I have been left behind with such...deep, deep despair and, uh...crying after being called the most insane things by my teenage daughter and her brother, who is a little younger.

The way the participants related to the videos depended on their experiences with similar situations. Elliot criticized the video of Erik's morning routine, based on a personal comparison.

The characters in the videos were portrayed by actors that followed an agreed-upon script. As such, the videos communicated a certain understanding of what it means to have ADHD. One participant wrote about the first video with Nora:

I do not identify with the character; I get the feeling she was chosen to “look like” she has ADHD. However, some parts of what she said were more relatable.

*P* #15

The participant distinguished between what is shown and what is said. The use of quotation marks can be interpreted as an ironic remark, and, therefore, such a depiction (ie, to “look like” having ADHD) is an inconceivable feat to truly accomplish. Looking like something includes imitating the mannerisms supposedly associated with it.

By the third module, the same participant had now changed their mind about whether Nora as a character was relatable:

This time, the video with Nora was “spot on.” It is definitely like me.

*P* #15

They went on to describe how Nora’s postponing chores was relatable. Here, the participant qualified the character of Nora as realistic because what she does is what the participant does. However, the participant did not revisit the previous theme of “looking like ADHD.” Elliot, who said the videos did not depict their experiences with ADHD accurately, was much more positively inclined to how Erik represents a “person with ADHD”:

He is like many, many others with ADHD. He is a dude that lives a family life and tries to handle his shit. He is not a caricature. He is...a normal dude, and that is nice.

Overall, the participants did not report a feeling of being misrepresented as a caricature of people with ADHD, except for participant #15 who gave a statement that could be interpreted in that direction.

#### Theme 2: Relating to the Characters and Their Situations

In this theme, we take a closer look at how the participants related socially and emotionally to the characters and their situations.

Alex said that the videos showing how to apply the techniques in corresponding situations were motivating:

I see that they can cope or make a change. [...] It inspires and motivates me to use the techniques shown in the videos, and the others

that are not covered in videos

Furthermore, Alex said that the videos show how the techniques are meant to be used:

Of course, I could have made the connections [between the technique and their intended outcomes] myself, but there is something extra with those videos: You get to see

how it is done

Thus, by setting an example of how to use a technique in the videos, the participants further understood the application of the technique in a certain context.

The video in which Nora is distracted during housework by a phone call from a friend who needed help purchasing a new dress (see example 2 in the Phase 1 Results section) received varied interpretations by the participants. Dylan speculated that Nora’s decision to go shopping with her friend was an act of procrastination because shopping with a friend is generally a more attractive option than housework:

It's a lot more fun to be with your friend, [...] it is really just that simple, that you do what is fun, right? But then things are constantly postponed, and then you come home, like Nora, to a messy apartment.

On the contrary, another participant related personally to Nora’s video, having a bad conscience when putting her needs before an important task:

[I] see that personality also plays a role here. [To what extent] do you get a bad conscience for your friend if you postpone or drop her [need for help]? I, myself, have had to do a major cleanup in my head over the past year to make more room for myself and my chores. It feels a little selfish and it is hard. But necessary.

P. #81

Similarly, Dylan explained the turmoil that postponing chores can lead to:

And then, for me, for example, that's when the negative self-talk begins: “That is what you should have done.” And then, you start cleaning, and then, it becomes a very big thing, a very negative thing.

The participants emphasized different aspects of the videos that made sense in relation to their own experiences in social situations where dilemmas can create feelings of uneasiness. Participant #81 and Dylan agreed that Nora represents how the negative outcomes of the dilemma commonly are turned inwards, either towards the self as feelings of guilt (#81) or as the negative self-talk described by Dylan. The participants showed empathy with the characters and used the situation in the video to contemplate their own experiences with everyday challenges and self-stigma.

We now turn to how the videos aroused negative feelings and recollections of past experiences in some of the participants. Alex found similarities between his or her own life and Erik’s morning routine in the videos for Module 2 (Breathe):

It is a situation I want to handle better. I should be able to do it, a few minutes for myself without an audio file, where I could breathe and relax the way he did in the video. I have not managed that yet, because I do not remember to do it.

Alex saw that Erik handled the situations in a better way because he had internalized techniques for controlled breathing to alleviate stress. Alex, on the other hand, said that they “should be able to do it,” but has not yet been able to implement the technique in everyday life. Alex needed a reminder for the breathing exercise, which seemed to be disappointing in comparison to the character Erik’s apparent mastered application of the technique.

In Module 6 (Acceptance), Erik is in a video conference planning a party with his friends. Erik loses focus and, when asked a question, becomes agitated and slams the laptop computer shut. Participant #62 wrote in the evaluation of this module:

[It] is useful to have a reminder to be kind to yourself. [I] realize that I still have a way to go when it comes to self-acceptance.

However, in the evaluation of the videos, the participant stated that “I thought this video was painful to watch, I cried afterward.” In the posttrial evaluation of the entire intervention program, the participant wrote that “[...] I could not complete the module [on acceptance] after the first video.” The negative experience with the video impeded the completion of the module, although this participant had identified it as useful. Unfortunately, we lack additional information on the participant’s experience of the situation. However, this example of adverse effects shows how videos that depict sensitive issues can be painful for participants that have lived such issues.

In this theme, we have seen how the participants reacted to the videos, sometimes by showing empathy with the characters and other times reflecting on their similar challenges.

#### Theme 3: Video Protagonists as Companions and Role Models for Change

The videos were motivating for the participants when there was a certain agreement between the depiction of ADHD in the video and the participant’s own experiences. In this theme, we explore how some participants related to the protagonists as role models for change.

One participant wrote the following as feedback to the videos that were shown in Module 6 (Acceptance):

For me, the videos are the best! At the same time, it hurts. But they show an educated, well-versed lady who struggles with the same things as me, and this is motivating.

*P* #50

Here, the participant described how they were motivated by the protagonist Nora, despite the videos provoking negative feelings. For participant #50, Nora impressed and motivated them. The protagonist appeared to be a “educated, well-versed lady” at the same time as she struggled with relatable challenges in everyday life.

The protagonists in the video served as role models for the motivation of the participants. Participant #81 found it helpful to see that others had similar thoughts and actions and used Nora as starting point for a reflection on their present situation:

Nora says that she will be better at using her time more constructively. [And] she will not be impatient if she does not see results right away. It is the same with me. [...] [I] do not have everyday routines yet. [I have a] new job, a new place to live, and I am looking for a romantic partner. So, making routines can be a bit of a challenge now.

Even though the participant does not explain how Nora influenced their perspective, their attitude toward their own life is influenced by knowing Nora as a character.

Participant #60 further elaborated on Nora being a role model, in the posttrial evaluation of the intervention program:

My biggest challenge in everyday life is postponing boring things, and it was precisely the video with Nora where I recognized myself. I set goals in everyday life. Thus, I have pushed myself to do all these routine chores better than before. I tidy up the apartment more often. The dirty dishes do not stay that long; instead, I clean them daily. I do the laundry, hung it up. This has been better almost throughout the training program.

It is striking how the participant’s reported improvements on everyday chores mirrored the challenges that Nora faces in the videos. The way that the participants addressed the protagonists shows that the videos contributed a social component to the intervention program.

Another social response to watching the videos is reflected in the report of feeling less alone:

It is good to watch the videos; it gives a feeling of not being alone when struggling with various things. And it gives me a sense of mastery in knowing I can do a lot of things that others are struggling with.

*P* #19

Alex found comfort in the videos:

I was looking forward to the movie clips because they felt so familiar. I saw myself, and it was amazing — what can I say? — good to feel that others are like that too.

Dylan, who struggled with feelings of low self-esteem and mental turmoil, found comfort in learning that others may feel the same way:

It has actually been a huge relief to find out that there are others who have it just like you; you are not as unique as you thought!

The experience of feeling less alone when struggling with everyday life was a relief for the participants. They learned about what it was like to have ADHD as an adult through participating in the intervention. For example, Dylan had little knowledge about ADHD, other than receiving the diagnosis and medication from their local psychiatric clinic, before participating. The study and the videos taught Dylan that their struggles with self-esteem and negative self-talk were something that others also experience.

Finally, participant #74 wrote in the posttrial evaluation that seeing the characters live ordinary lives was a positive aspect of the intervention program:

Seeing other adults with ADHD, who live ordinary lives, in the videos, has been very nice, and [...] the focus on the fact that there is a lot of positive [aspects of] the diagnosis. I live a very good life myself

emoji: Heart

## Discussion

### Principal Findings

#### Overview

In this study, we addressed the design of therapeutic content, in particular video vignettes, for adults with ADHD founded on the experiences of their peers. It was carried out as a participatory design study, followed by qualitative feedback from participants in a clinical trial of an online intervention.

Our findings show that the video vignettes’ depiction of everyday situations resonated with the participants and, as a result, reflected their experiences. The participants recognized the situations and used the characters as role models for change.

#### Maintaining Authenticity in Narratives for Online Interventions

Personal narratives on social media have become an increasingly important and popular source of information about physical [46-48] and mental health [44,51]. Here, we discuss how videos based on personal narratives were used to create engaging content for the MyADHD intervention. We further discuss the trade-offs between maintaining authenticity and stylizing characteristics of mental health and how they affect stigmatization.

The use of video vignettes was an engaging feature of the MyADHD intervention, and by producing our videos, we could ensure that the content would abide by the therapeutic principles. Video as a design material offers diverse ways to contextualize narratives. For example, the choice of actors, location, set decoration, editing, and general style of the video lay the foundations for the viewer’s experience with the content. However, in our intervention, the protagonist Nora seemed to be “chosen to ‘look like’ she has ADHD,” as reported in theme 1 in the thematic analysis. This remark is timely because ADHD is associated with public stigma, often perpetuated in media reports [31-34]. In the video vignettes of MyADHD, the actors who portrayed the protagonists acted as if they had an ADHD diagnosis. The actors’ performance can come off as a caricature that, in turn, reinforces self-stigmatizing beliefs in people of the target group or may cause skepticism about the authenticity of the narrative. Perhaps the credibility of the narrative could be better maintained if the video vignettes followed people with ADHD. A documentary style might resemble more the way health vloggers share their experiences with diagnosis and illness [46-48] and have been utilized in the studies by Amsalem et al [57,58], using people with the lived experience of schizophrenia in videos to reduce public stigma.

Honary et al [49] found that using actors as “talking heads” representing the lived experience of mental illness in videos was important for the protection of the identity and anonymity of the participants in their study. However, in some cases such as interventions for groups that experience public stigma, active participation in the content may be a meaningful and empowering activity for the participants. Designers of interventions for people with mental disorders could explore the possibility of the participants participating in video vignettes narrating their everyday life, provided they are motivated and informed about the implications of such exposure. Representatives of the target group are excellent communicators of authentic experiences of mental health issues in everyday life. Such participation, however, requires careful considerations of the ethical implications. Whereas sharing personal experiences of mental health on social media is a private initiative, to disclose a person’s mental health in the context of a research study is essentially a public matter. Research-led disclosure would require a thorough assessment and follow-up of the participants’ safety.

Based on our thematic analysis, we found that the participants perceived the protagonists Nora and Erik as characters. The participants related to them as examples of how ADHD symptoms may manifest in everyday life while being aware of the boundaries of the characters and understanding that they do not necessarily generalize the ADHD experience. Looking like someone with ADHD, however, can take many forms since people with ADHD represent a heterogeneous group with diverse characteristics and experiences. For example, the video content designed in this study is limited to a dichotomy between male and female ADHD. However, people’s gender identities are known to be fluid, implying that inclusive intervention content is needed. Future research should explore how we represent the diversity of mental health in online interventions. For example, including people with multiple marginalized identities as reported in former studies (eg, [71]) is an exciting possibility in the processes of designing more diverse and inclusive online interventions.

In designing video vignettes that apply narratives of the everyday lives of people with mental health disorders, designers must be aware of the trade-off between preserving the authenticity and reinforcing stigmatizing characteristics of mental health. Using actors protects the identities of people with mental health problems, while, on the other hand, affects the authenticity and may contribute to stigmatizing caricatures of the experience of mental health.

#### Grounding Therapeutic Content in Everyday Life Situations

Online clinical health communication has been described as fact-based and prescriptive [53]. In our experience, this conceptualization is transferable to the communication of therapeutic content in psychological online interventions. In this part, we discuss how we have explicitly focused on everyday situations to help adults with ADHD integrate the MyADHD intervention in their daily lives. We emphasize the importance of striking a balance between the experiential, lived perspective of mental health with the more prescriptive, clinical presentation of how to self-manage mental health when designing video vignettes.

In online interventions, exercises and coping techniques from CBT, DBT, and other therapies are presented through text, audio, or video; they guide the participant in successfully applying these techniques and self-managing their mental health. However, in the design of unguided online interventions, it is particularly important to consider how the techniques are presented. In this case, the absence of a therapeutic relationship places greater demands on the design of the therapeutic content. Exercises and techniques can be difficult to understand as they are intended. They presuppose the understanding of both the application and the relevance to one’s everyday life. For the intervention to be effective and meaningful, the exercises and coping techniques should be exemplified by everyday relatable situations. By doing so, the techniques can be transferable and implemented into day-to-day challenging situations. Seeing peers cope with challenging situations may enhance one’s sense of self-efficacy [54,55].

The video vignettes in MyADHD offer contextualization of difficult everyday situations in scenarios recognizable and relatable to adults with ADHD, based on their own experiences. For example, in the first video for Module 2 (Breathe; see Table S1, Multimedia Appendix 5), the protagonist, Erik, was overwhelmed with stress by the morning routine with the family. In the follow-up video, Erik applied a coping technique to prepare himself before getting on with the morning routine and, thus, had a less stressful morning with his family. In this way, the videos contextualized psychological coping techniques by showing their intended use and outcomes in a variety of everyday situations. We established the psychological coping techniques using examples of mundane everyday situations. Thus, we followed Feuston and Piper’s [51] focus on the lived experience of mental health through an everyday lens. By grounding the therapeutic content in the daily struggles, we sought to challenge sensationalized [32,34] and stigmatizing [35,37] narratives of what it means to live with ADHD.

The video vignettes portrayed people with ADHD through characters that held steady jobs, good relationships, and meaningful everyday lives. In this way, we tried to formulate an alternative to didactic, theory-driven narrations of therapeutic content. By providing a clear context to both the symptoms of and coping techniques for ADHD-related challenges, we sought to offer a nonsensationalized view of the disorder that resonated with our participants. In theme 3 of the phase 2 findings, we analyzed how the participants were inspired by the character Nora’s actions. The participants’ reports of feeling motivated when seeing the characters coping with ADHD-related challenges could be seen in light of Bandura’s [54,55] social cognitive theory, where observing role models is essential to facilitate learning. However, the videos might be less relatable and motivating when they show the coping techniques perfectly applied, especially to those that know how difficult those efforts can be. This is exemplified by Elliot’s statement in theme 1 of the phase 2 findings: According to the participant, the videos “fell flat” regarding the depiction of everyday life with ADHD. This shortcoming can be explained by the context (ie, an online intervention with therapeutic aims) or the participatory process of designing the video vignettes.

Blandford et al [10] addressed how health and HCI research understand user needs from diametrically opposing positions: from a theoretical and evidence-based viewpoint (top-down) [72] and from consulting or involving users directly as participants in design activities (bottom-up) [63], respectively. For therapeutic content to be properly grounded in the everyday experiences of the target group, a bottom-up approach is required. Involving participants in design activities permits the creation of realistic and relatable scenarios. However, it is necessary to be conscious of the activity one is designing for, which, in this study, was an online unguided therapy aimed to improve the management of everyday challenges for adults with ADHD. The design of content that is both effective in mediating therapy as well as relevant and consistent with the target group’s experiences and values must be guided by both therapeutic aims and an experiential perspective on mental health. This, in the context of online interventions, requires a balance between the sometimes conflicting prescriptive and experiential views on mental health.

#### Configuring Participatory Design of Intervention Content

Here, we discuss how participatory design can complement and extend specialized approaches to the design of online interventions to align the content with the values and experiences of the target groups. Furthermore, we discuss how the participatory process applied here could be adapted for the design of online interventions destined for other target groups.

Designing narratives of mental health as content in an online intervention requires understanding mental health from an experiential perspective. Therefore, careful consideration of representing the diversity of experiences and identities of the target group is necessary to be inclusive of a variety of identities. Here, we argue that a participatory design process that includes people with first-hand experiences of relevant mental health issues is an appropriate method. Participatory design can include the voices of people with a breadth of experiences and help strike a balance between the lived experience and the therapeutic expertise of clinicians.

The MyADHD intervention was developed following the person-based approach [4]. Although this approach is partly rooted in user-centered design methods, Yardley et al [4] explicitly differentiated this approach from participatory approaches that include co-design activities:

A potential problem with [the co-design] approach is that it encourages users to try to anticipate the needs of others, which they are unlikely to do well, rather than simply reporting their own experiences and views, which they do very well. We find that users are naturally expert at telling us what they like or dislike about our intervention, but most users are understandably less able to generate effective behavior change techniques or good design solutions.

In the development of MyADHD, however, we used the co-design method of participatory design to specifically engage people with ADHD and make their voices heard in the process of designing therapeutic content. We found that the participants were not just experts on “what they like or dislike,” but intimately knowledgeable of ADHD as it is lived by their first-hand experience and tacit knowledge of being diagnosed with ADHD. Building on their knowledge, we designed content that represented the participants of the intervention study. So, we have constructively extended the person-based approach by including participatory design in the process of designing content for the *MyADHD* intervention.

In this paper, we showed that the core therapeutic aims defined by clinicians are retained while, at the same time, people’s experiences with ADHD are directly included in an online intervention through participatory design. According to Yardley et al [4], participants are “less able” to generate behavior change techniques. It has been recommended that the behavior change techniques of online interventions should be based on sound theory and evidence to substantiate their effect [4,72,73]. In this study, the participants primarily contributed to co-designing the presentation of behavior change techniques. In the ideation workshop, participants did, however, also contribute by suggesting coping strategies that they used to manage their everyday lives (see Table S2, Multimedia Appendix 5 for a diverse selection of suggestions). It is our position from conducting this study that participants can be *enabled* to contribute to the design of behavior change techniques — this is a matter of proper design process facilitation.

According to Bratteteig and Wagner [63], participatory design “may have to operate in a highly structured environment that imposes particular ‘rules’ and surely it has to define its own ways of operating” [63]. Referring to this, we took a pragmatic position: The clinicians defined the clinical needs and the treatment outcomes according to theoretical models and their expertise. In this study, the use of principles and techniques from psychological interventions coupled with psychoeducation enhanced the perceived mastery of everyday life challenges for adults with ADHD. Experts by experience contributed by narrating their everyday life and were, thus, in a position to share “what the user wants.” Here, it was a request to use videos to narrate content that the participants not only suggested but also took part in designing.

The design process led to mutual learning among the parties (ie, clinicians, participants, and the film production team) involved in the design of the video vignettes. This learning was developed within meetings, workshops, and film production sets. For example, clinicians would learn about specific difficult situations for the adults, whereas the participants learned how their experiences fit within a clinical viewpoint and boundaries for the management of ADHD symptoms. The film production team conceptualized these ideas into a video format. The video scripts would then be reworked under the other parties’ perspectives. The presentation of the video vignettes was further enhanced as engaging content by the skillful execution of the film production team. The video vignettes were, in a sense, grounded in the tacit knowledge [61] of the participatory design participants. The lived experience of having an ADHD diagnosis was not available to the clinicians initially; the participants brought their experiences into the process through the mutual learning facilitated by the participatory design process.

The design process of making video vignettes for the MyADHD was costly and time-intensive: It required resources for a film production team that would realize the vision of the participatory design process. However, we encourage the application of our process in the development of psychological online interventions that aim to create therapeutic content grounded in everyday contexts for their target groups. Similar to how health vloggers produce technically simple productions with consumer products (eg, smartphones, web cameras), designers of content for psychological interventions can do the same.

We suggest that resonance with the participant’s values and experiences is vital for this kind of content to be perceived as meaningful and further suggest that researchers and designers of online interventions should strive to achieve this in their efforts to produce narrative content that supports therapeutic principles.

Person-centered approaches to design have been emphasized as essential to create content that is adapted to the target group’s shared values and experiences [8,9]. In this regard, we found the co-design activities of the participatory design method complemented the person-based approach in developing online interventions. The participatory design approach comes from a democratic perspective of giving future users a say in the design of information technology solutions [61,62] and, therefore, can be used to align the intervention with the values, experiences, and needs of potential adopters. However, for the designed content to be successful in supporting therapeutic principles, the participatory design for online interventions needs to be carefully facilitated. We recommend that researchers and designers of online interventions adapt co-design to their target group and specific purpose — there is no one-size-fits-all approach to meaningful co-design.

Regarding the involvement of people with ADHD in our process, we found, contrary to Yardley et al [4], that the “users” were indeed competent in generating adequate design solutions. In our experience, the participants contributed with interest and determination in designing creative, novel narrative content. Their contributions possessed qualities in ways we could not have anticipated. We found their involvement in this study decisive in designing content that represented the target group’s values and experiences.

### Limitations

This qualitative study has limitations. First, in the clinical trial and the follow-up interviews, women were overrepresented compared to men. Thus, our findings may not be representative of men with ADHD. Second, the ADHD diagnosis was self-reported. Last, this is a qualitative study and thus does not ascertain the videos’ or the intervention’s clinical effects. Further details of the study design and dissemination of the effects and clinical outcomes will be reported in a separate publication.

### Conclusions

In this paper, we presented our process for designing video vignettes portraying challenging situations of living with ADHD, with the participation of and directed to adults with ADHD in a therapeutic context. Based on our findings, the approach of using an everyday lens and describing ostensibly mundane and everyday contexts in the videos was well-received by the participants in a clinical trial of an unguided online intervention. The videos provided rich and contextualized illustrations of life with ADHD, designed beyond the need for self-management in core symptoms. Applying a participatory approach when designing for online interventions, however, requires a balance between the lived experience as reported by the participants and the therapeutic expertise of the clinicians to be relevant to the target group.

## Appendix

Multimedia Appendix 1 This is the first video of Module 2: Breathe where we are presented with Erik and his problems with morning routines.

Multimedia Appendix 2 This is the second video of Module 2: Breathe, where we see Erik handle his family's morning routines better now that he is applying a technique taught in the MyADHD intervention.

Multimedia Appendix 3 In Module 3: Stop, we meet Nora who is struggling to maintain focus on completing everyday chores.

Multimedia Appendix 4 In this video, from Module 3: Stop, we see how Nora handles interruptions in her everyday chores by applying a "Stop"-technique.

Multimedia Appendix 5 Video vignettes designed for the MyADHD intervention.

Multimedia Appendix 6 A selection of excerpts from Ideation Workshop in Phase One. Quotes are from clinicians' notes. The examples stylized in italics were used directly in the video vignettes.

## Abbreviations

ADHD: attention deficit hyperactivity disorder
ASRS: Adult ADHD Self-Report Scale
CBT: cognitive behavioral therapy
DBT: dialectical behavioral therapy
HCI: human-computer interaction
