# Supplementary material for: Designing Videos With and for Adults With ADHD for an Online Intervention: Participatory Design Study and Thematic Analysis of Evaluation
Source: JMIR Ment Health. 2021 Sep 14;8(9):e30292. doi: 10.2196/30292 (PMC8479608; doi:10.2196/30292)
Supplement: Multimedia Appendix 5 [file mental_v8i9e30292_app5.docx]

Table S1. Video vignettes designed for the *MyADHD* intervention.

| Module | Protagonist | Description | Exercise | Duration |
| --- | --- | --- | --- | --- |
| Recruitment site | Nora | A day in the life of Nora, as narrated by her to her therapist. | Various | 02:58 |
| 1: Intro | Nora | A monologue explaining how ADHD has a positive influence on Nora. Examples of how inattention impacts her everyday life. | N/A | 03:47 |
| 2: Breathe | Erik | (1) Morning routine with the family and how it leads to stressful situations. (2) Using an exercise of controlled breathing helps Erik to calm down in the morning. | Controlled breathing | 01:02 & 00:49 |
| 3: Stop | Nora | (1) Everyday chores delayed until the last minute. (2) The stop-technique helps Nora prioritize chores over leisure activities. | Stop | 01:23 & 00:45 |
| 4: Emotions | Erik | (1) Erik becomes agitated by a non-functioning dishwasher and mouths off to his wife. (2) Erik stops to breathe as he becomes agitated, which helps him regain emotional control. | Square breathing | 00:36 & 00:26 |
| 5: Planning | Nora | (1.1) Nora spends her day at work procrastinating when she should have completed a financial report. She works overtime. (1.2) Nora reflects on why she did not get things done. (2.1) Nora works overtime from home, is repeatedly distracted by other activities, and finishes work late at night in bed. (2.2) Nora receives an auditory notification on her phone from a social media app but quickly turns off notification sounds and resumes work. We follow Nora as she completes tasks from her to-do list. | Making lists and breaking down tasks into sub-tasks | (1) 03:30 & 02:25 (2) 01:15 & 00:46 |
| 6: Acceptance | Erik | (1) Making plans with friends. Erik loses focus and becomes agitated and shameful when everybody notices. (2) He accepts the mishappening when noticed, addresses it, and continues the planning. | Self-acceptance | 01:01 & 01:00 |
