# Supplementary material for: Designing Videos With and for Adults With ADHD for an Online Intervention: Participatory Design Study and Thematic Analysis of Evaluation
Source: JMIR Ment Health. 2021 Sep 14;8(9):e30292. doi: 10.2196/30292 (PMC8479608; doi:10.2196/30292)
Supplement: Multimedia Appendix 6 [file mental_v8i9e30292_app6.docx]

Table S2. A selection of excerpts from Ideation Workshop in Phase One. Quotes are from clinicians' notes. The examples stylized in italics were used directly in the video vignettes.

|  | **Experiences and examples related to topics** | **Coping strategies suggested by participants** |
| --- | --- | --- |
| Module 2: «Breathe» | - Participants did not have any concrete examples of situations where they saw the benefit of mindfulness or breathing exercises - Clinicians chose the following example for this module’s video vignette put forward by participants in the discussion of other topics:   - *“You notice challenges related to ADHD more strongly after you have children, then you are responsible for more than yourself. One must take care of everyone else as well.”*   - *“It can be helpful to stop in the morning routine; you have to remember a lot of things before you leave home, especially when you have children for whom you are responsible. It is extra stressful when there is so much to remember for the children as well, and it can quickly become quite chaotic, especially in the morning.”* | - Breathing exercises/mindfulness seen as useful in stressful situations and difficult life situations. - Some participants said that mindfulness exercises are hard to follow and to maintain focus throughout guided exercises. |
| Module 3: «Stop» | - “It is important to take a moment to “stop” when you do not have deadlines from others, because then there is no external pressure on you to actually finish the goals you have set.” - “Take my mobile phone apart or tinker with the TV when I have to do other things. Can sit and fiddle for far too long before I can stop.” - “A confusing or stressful situation, it is easy to 'put the pedal to the metal’. It is like driving a car and accelerating when there are difficult conditions. Then you really should slow down!” - *“For example, using the duster. I go and get it, but then I think of something else to do. I then leave the house, come back much later, and have not gotten any chores done”* | - “A list of specific goals, an overview of what needs to be done, and can thus monitor that you are on the right path.” - “Divide tasks into smaller sub-goals; it helps if things are confusing, and you do not know where to start.” - “Write down appointments earlier than they really are, so that you have a little extra time to go in case you should be too late for an appointment.” |
| Module 4: «Emotions» | - “Sometimes you experience that you keep a lot of frustration inside you that builds up - until you explode. For example, to experience a difficult situation at work, you can also not react there and then, but then everything comes out when you come home.” - *Experiences that others do not understand their feelings, people around think that they are upset for “nothing” and that their reactions are excessive. Feeling like a burden to those around due to strong emotional reactions, and often experience feelings of remorse and guilt afterwards.* - “Hard to stop once you are in the situation. I kind of become like The Hulk" | - “Write down what I feel and why I feel that way” - “Talk about it with others. If you cannot talk to others immediately, then you can move away from the situation and for example go to the bathroom and write down a note on your mobile there” - “Hit pillows or other harmless things” |
| Module 5: «Planning» | - *“You are at work and unable to complete the work tasks. You spend more time helping others than completing your own tasks. Colleagues come to ask for help, and then you spend too much time being helpful, and thus forget your own work tasks.”* - “I can make lots of plans–no problem! But there is no point in making plans if they are not executed.” | - Fixed routines for everyday life - Get away from the situation or get away from what distracts oneself and prevents one from achieving a goal. |
| Module 6: «Acceptance» | - “In many situations, one thinks that others think one is weird” - “Sometimes you work so hard to be "normal" that you only look at everything you do wrong” - In social situations: “I have to work so hard at keeping my mouth shut while eating that I hardly get to enjoy the food” - “If I make a mistake several times in a row, for example that for the fifth time this week I have to turn the car on my way to work to get the access card, then I may get very annoyed with myself and say ugly things about myself.” - “When you have children, you have to stop thinking about your own needs. It makes you extra tired when you have children. Where am I in all this?” - “I hear that critical voice going ‘now you’ve done it again, you are hopeless’” | - “Accept that one has some difficulties that others do not have” - “Talk with others who have ADHD” - “Boasting of oneself when one achieves goals” - About accepting that one has forgot their access card: “It is very dependent on context, other times I just think ‘shit happens’” - “Think of having ADHD as an entry into the ‘ADHD family’” - “Remind oneself that others also need your acceptance” - “It is easier to accept myself now that I have a diagnosis. Now I have an explanation for my behavior” - “Do like Britney Spears, say to yourself ‘Oops, I did it again!’ With that, things are less serious but also more fun!” - “Allow yourself to hate the dishwasher. I hate it. I can swear as much as I want, no shame, and I will do it as I please” |
